# An Artificial Light Source Influences Mating and Oviposition of Black Soldier Flies, *Hermetia illucens*

**DOI:** 10.1673/031.010.20201

**Published:** 2010-12-03

**Authors:** Jibin Zhang, Ling Huang, Jin He, Jeffery K. Tomberlin, Jianhong Li, Chaoliang Lei, Ming Sun, Ziduo Liu, Ziniu Yu

**Affiliations:** ^1^ State Key Laboratory of Agricultural Microbiology, National Engineering Research Centre of Microbial Pesticides, Huazhong Agricultural University, Wuhan 430070, P. R. China; ^2^ Department of Entomology, 2475 Texas A&M University, College Station, TX 77843–2475, USA; ^3^ Department of Entomology, Huazhong Agricultural University, Wuhan 430070, P.R. China

**Keywords:** development, manure management, mass-rearing, quartz-iodine lamp

## Abstract

Current methods for mass-rearing black soldier flies, *Hermetia illucens* (L.) (Diptera: Stratiomyidae), in the laboratory are dependent on sunlight. Quartz-iodine lamps and rare earth lamps were examined as artificial light sources for stimulating *H. illucens* to mate and lay eggs. Sunlight was used as the control. Adults in the quartz-iodine lamp treatment had a mating rate of 61% of those in the sunlight control. No mating occurred when the rare earth lamp was used as a substitute. Egg hatch for the quartz-iodine lamp and sunlight treatments occurred in approximately 4 days, and the hatch rate was similar between these two treatments. Larval and pupal development under these treatments required approximately 18 and 15 days at 28°° C, respectively. Development of methods for mass rearing of *H. illucens* using artificial light will enable production of this fly throughout the year without investing in greenhouse space or requiring sunlight.

## Introduction

The black soldier fly, *Hermetia illucens* (L.) (Diptera: Stratiomyidae), is a large wasp-like fly distributed throughout tropical and temperate regions. It produces three generations per year from April to November in the southeastern region of the United States (Sheppard et al. 1994) with larvae occurring in very dense populations in organic wastes such as manure (Sheppard 1983), coffee bean pulp (Lardéé 1989), and carrion (Tomberlin et al. 2005).

The black soldier fly is useful for managing large concentrations of animal manure and other organic waste. Its larvae can reduce manure accumulation by 50% (Sheppard 1983; Meyers et al. 2008), including a 24% reduction in nitrogen concentration (Sheppard et al. 1994); and they can also reduce the amount of *Escherichia coli* in dairy manure (Erickson et al. 2004; Liu et al. 2008). Prepupae are composed of 42% crude protein, 35% fat (Sheppard et al. 1994), and contain essential amino acids. If a source such as fish offal is included in their diet the prepupae can accumulate significant levels of omega-3 and essential unsaturated fatty acids such as α?-linolenic acid (ALA), eicosapentaenoic acid (EPA), and docosahexaenoic acid (DHA) (St-Hilaire et al. 2007). When dried for use as feedstuff, the prepupae have an estimated value comparable to soybean or meat and bone meal. Their value as a product might be higher if they were to be used live as a special feed type, or if they were marketed to promote their other unique qualities such as essential fatty acids and chitin (Sheppard et al. 1994; Tomberlin et al. 2002b).

*H. illucens* is not active in the winter months in most of the world, and this necessitates their being reared indoors under artificial conditions. Tingle et al. (1975) reported *H. illucens* mating and ovipositing ““often”” in a 3 ×× 6.1 ×× 1.8 m cage that was kept outdoors because direct sunlight is known to encourage mating in *H. illucens* (Cribb 2000; Tomberlin et al. 2002a). In fact, few mating pairs were observed during the winter or on cloudy days because of the lack of exposure to sunlight (Tomberlin et al. 2002a). Previously, a 40-watt Sylvania Gro Lux®® and a 430-watt Pro Ultralight Light System®® were found to be unsuccessful in eliciting mating behavior (Tomberlin and Sheppard 2002a). An artificial light source capable of eliciting mating would facilitate mass production and utilization of this beneficial arthropod outside of their typical activity patterns and in non-native regions (Sheppard et al. 2002). The objective of this study was to determine if an artificial light source could stimulate the mating of *H. illucens,* thereby overcoming the dependency on sunlight for colony maintenance of this species.

## Materials and Methods

### Source of flies

An *H. illucens* colony was maintained at the National Engineering Research Centre of Microbial Pesticides, Huazhong Agricultural University, Wuhan, Hubei, China.

### Light sources

A 500-watt quartz-iodine lamp (Model QVF135, Philips Lighting Ltd., www.lighting.phillips.com) with a spectrum between 350–2500 nm and a 450-watt rareearth light (Engineering University Infrared Technology Research institute, Harbin, Heilung- kiang, China) with a spectrum between 350–450 nm were used as experimental treatments. Natural sunlight was used as the control. During the experiments sunrise was at approximately 05:30, and sunset was at 18:00 in Wuhan, Hubei, China. The artificial light sources were turned on at 08:00 and turned off at 17:00 daily until all of the adults had died. A Quantum Meter (Apogee Instruments Inc., www.apogeeinstruments.com) was used to measure the light intensity (μ?mol m-2s-1) of visible light (400 nm and 700 nm) during each observation period. All light readings were taken at 50 cm below the bulb.

### Experimental design

Three 1.8 ×× 1.2 ×× 1.5 m cages were used. One cage was placed in a greenhouse where sunlight and adequate space for aerial mating were available. Two more cages were covered with white screens and used for the artificial light treatments. These cages were placed in a room without sunlight. The quartz-iodine and rare-earth lights were suspended approximately 10 cm above the cages. For each light treatment, approximately 1000 newly-emerged adult *H. illucens* from the colony were released into each cage. During the experiments, new adults were added on two occasions to the cages receiving artificial light and sunlight to maintain the cage population at approximately 1000 individuals. The temperature and RH were maintained at 28°° C and 60%, respectively. All experiments were carried out between October 2007 and December 2008. The quartz-iodine and rareearth light treatments were replicated consecutively on three occasions. The sunlight treatment was conducted on three occasions for comparison purposes with both artificial light treatments.

### Counts of the number of mating pairs

Counts of the number of mating pairs were initiated on the date that *H. illucens* were released into the cages. Observations were made for 10 min periods at 1 h intervals during daylight hours (9 h) each day

### Measurement of oviposition rates

The rate of oviposition was determined by recording egg clutches deposited daily. To make this measurement, a plastic pot with a diameter of 25 cm ×× depth of 10 cm containing 1 kg of a saturated grain diet of 50% wheat bran, 30% alfalfa meal, and 20% corn meal (Hogsette 1992) was placed in the center of the cage on a table at a height of 40 cm. Individuals oviposited in the flutes (2 ×× 3 mm) of two corrugated cardboard rolls (egg collecting units) measuring 2.5 cm in diameter ×× 3.5 cm in length that were taped to the inside of the pot approximately 3 cm above the moist media. The cardboard rolls were replaced daily, and the numbers of egg clutches were recorded.

### Influence of light on immature stages

To compare immature life-history traits between the sunlight and artificial light treatments, the cardboard rolls containing eggs were placed in different 0.5-L glass containers. Each container was covered with a paper towel that was held in place with a rubber band and stored in a greenhouse at 28°° C and 60% RH until larvae emerged. The larvae were reared using a saturated grain diet (Hogsette 1992). To differentiate life stages, prepupae had a characteristic black cuticle whereas larvae were white, and larval development time was the time from the appearance of the first larvae to the appearance of the first prepupae. Pupal development included the time from the appearance of prepupae to the first adult emergence. The sample sizes were 100 for each cohort.

### Statistical Analysis

Larval and pupal development times were analyzed using PROC Mixed software (SAS Institute 1998, China). The least significant difference (LSD) test was used, if there was a significant value from an initial F test (*P* ≤? 0.05), to separate the mean differences for each variable.

## Results

**Figure 1.**
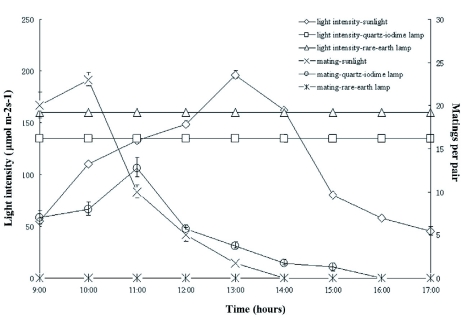
The influence of different light sources on the mating of *Hermetia illucens* and the number of pairs mating were recorded in one day. The experiment was replicated three times. High quality figures are available online.

Under sunlight, approximately 70 mating pairs were recorded daily (Figure 1). Mating began at 08:30 and peaked at 10:00 with an average of 23 mating pairs at a light intensity of approximately 110 μ? m^-2^s^-1^. Eightyfive percent of the mating activity occurred during the morning. Mating activity decreased when the light intensity was more than 110 μ?mol m^-2^s^-1^. On average, two mating pairs were observed at 13:00 when light intensity was 200 μ?mol m^-2^s^-1^.

Mating activity was not observed when using the rare-earth light. Under the quartz-iodine lamp, approximately 40 mating pairs were observed per day. This value was approximately 39% less than observed when observing the effects of sunlight (Figure 1).

The eggs collected from the sunlight and quartz-iodine lamp treatments were reared in the insectarium. There were no significant differences in the larval and pupal development times of the eggs collected from the two light sources (Table 1).

Mating and oviposition of the second and third cohorts were observed under the quartziodine lamp. Eggs collected from the second and third cohorts under the quartz-iodine lamp treatment hatched, pupated, and emerged. The larval and pupal development times were not significantly different among the three cohorts under the quartz-iodine lamp (P>0.05) (Table 2).

**Figure 2.**
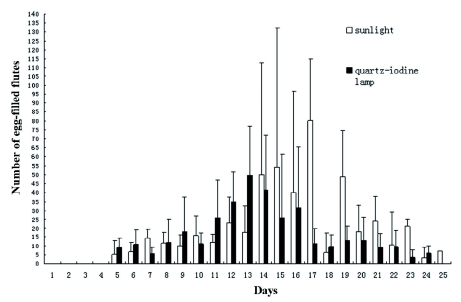
The amount of egg-filled flutes collected under the sunlight and the quartz-iodine lamp treatments in a cage during a 25 day period. The experiment was replicated three times. High quality figures are available online.

Oviposition peaked on the 17^th^ day when using sunlight (Figure 2). Under the quartziodine lamp treatment, a similar peak appeared on the 13^th^ day and lasted for 6 d with the highest level of egg production being approximately 61.9% of that observed in the sunlight treatment. There was no significant difference (P>0.05) in the number of egg masses laid between the sunlight and the quartz-iodine lamp treatments (Figure 2).

## Discussion

Previously, Tomberlin and Sheppard (2002a) reported that the intensity of sunlight plays a major role in determining when *H. illucens* will mate. They found that oviposition behavior was mediated by time of day. Largescale artificial rearing of *H. illucens* has sometimes been carried out with no sunlight, in which cases low mating and oviposition rates occurred. Tomberlin (2002a) used artificial lamps (such as a 430-watt Pro Ultralight light system and a 40-watt Sylvania Gro Lux system) to stimulate mating and oviposition, but no mating was observed. To test artificial light sources in stimulating mating and ovipositing, two lamps were used in this study, a quartz-iodine lamp and a rare earth lamp. The reason for choosing these two artificial light sources was that the spectrum of the quartz-iodine lamp is similar to that of sunlight, and the spectrum of the rare earth lamp is in the ultraviolet and violet light range of visible light; thus, insects could be attracted by either or both of these spectra.

**Table 1.**
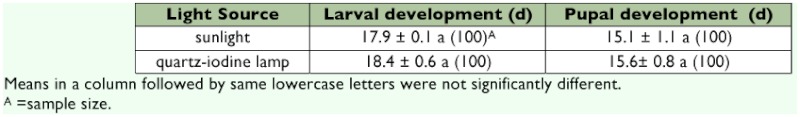
Immature Life-history traits of black soldier fly reared under sunlight and quartz-iodine lamps.

**Table 2.**
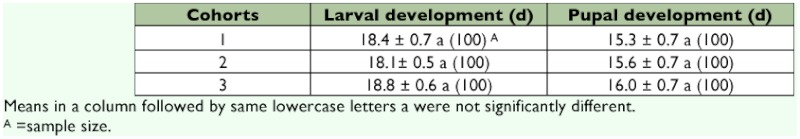
Immature Life-history traits of black soldier fly reared under quartz-iodine lamps

In this study, it was demonstrated that a 500watt, 135 μ?mol m^-2^s^-1^ light intensity quartziodine lamp could stimulate mating and oviposition, and the subsequent larval and pupal development times were comparable to results produced under natural sunlight. In contrast, the 160 μ?mol m^-2^s^-1^ light intensity rare-earth lamp did not stimulate mating. Tomberlin speculated that the eyes of male *H. illucens* might cue in to specific wavelengths of sunlight (Tomberlin and Sheppard 2002a).

Briscoe and Chittka (2001) indicated that insects normally cannot see red or infrared light. In fact, the longest wavelength that can be seen by insects is 700 nm. Therefore, light wavelengths from 700 to 2500 nm are not responsible for stimulating mating and oviposition. Furthermore, the results showed that a 350 to 450 nm wavelength rare earth lamp could not stimulate mating and ovipositing. Briscoe also reported that 12 of 16 species of Diptera that were examined had receptors for high blue or low green light (lambda max: 480–530 nm); thus, light in this range may be responsible for inducing mating activity. The results of our study suggest that wavelengths from 450 to 700 nm were influencing mating behavior. To better understand the biology of *H. illucens* and how infrared light influences its mating behavior, additional research is needed to select overlapping wavelengths and then to identify the suitable light spectrum for induction of successful mating. This study provides new information on mating and oviposition of *H. illucens* exposed to different light sources and demonstrates the successful use of artificial light to elicit mating in this insect. The results will be useful in improving current methods of *H. illucens* rearing during winter and under cloudy conditions.
